# Foliar application of abscisic acid mitigates cadmium stress and increases food safety of cadmium-sensitive lettuce (*Lactuca sativa* L.) genotype

**DOI:** 10.7717/peerj.9270

**Published:** 2020-07-02

**Authors:** Mohammed Mujitaba Dawuda, Weibiao Liao, Linli Hu, Jihua Yu, Jianming Xie, Alejandro Calderón-Urrea, Yue Wu, Zhongqi Tang

**Affiliations:** 1 College of Horticulture, Gansu Agricultural University, Lanzhou, China; 2 Department of Biology, California State University, Fresno, Fresno, CA, United States of America

**Keywords:** ABA-inhibitor, Antioxidant enzymes, Cadmium stress, Nutrient elements, Hydrogen peroxide, Malondialdehyde, Chlorophyll content, *Lactuca sativa*

## Abstract

Cadmium (Cd^2 +^) is among the toxic non-essential heavy metals that adversely affect plants metabolic processes and the safety of produce. However, plant hormones can improve plant’s tolerance to various stresses. This study investigated the effect of exogenous abscisic acid (ABA) on the biochemical and physiological processes and food safety of cadmium (Cd^2 +^)-sensitive lettuce genotype (*Lüsu*). Seedlings were subjected to five treatments: [(i) Control (untreated plants), (ii) 100 µM CdCl_2_, (iii) 100 µM CdCl_2_+10 µg L^−1^ ABA (iv) 10 µg L^−1^ ABA, and (v) 0.01 g L^−1^ ABA-inhibitor (fluridone)] for fourteen days in hydroponic system. The 100 µM CdCl_2_ increased the contents of hydrogen peroxide (H_2_O_2_) and malondialdehyde (MDA), decreased photosynthesis and plant biomass. Moreover, it decreased the contents of essential nutrients (except copper) in the leaves but increased the contents of toxic Cd^2 +^ in the leaves and roots of the plants. Foliar application of fluridone (0.01 g L^−1^) also caused oxidative stress by increasing the contents of H_2_O_2_ and MDA. It also decreased the contents of nutrient elements in the leaves of the plants. However, exogenous ABA (10 µg L^−1^) mitigated the Cd^2 +^-induced stress, increased antioxidant enzymes activities, photosynthesis and plant biomass under CdCl_2_ treatment. Remarkably, exogenous ABA increased the contents of essential nutrient elements but decreased the Cd^2 +^ content in leaves under the CdCl_2_ treatment. Our results have demonstrated that foliar application of ABA mitigates Cd^2 +^ stress and increases the nutritional quality and food safety of Cd^2 +^-sensitive lettuce genotype under CdCl_2_ treatment.

## Introduction

Cadmium (Cd^2+^) is a non-essential heavy metal, which is highly toxic and adversely affects several plant metabolic processes by causing oxidative stress (Gratão et al., 2015). The main cause of oxidative stress in plants is the increase production of reactive oxygen species (ROS) such as hydrogen peroxide (H_2_O_2_), superoxide anion (O_2_), singlet oxygen (^1^O_2_) and hydroxyl radical (-OH) in the cell (Sarvikas & Tyystjärvi, 2010). Although reactive oxygen species (ROS) are harmful to plant cells at higher concentrations, at normal levels, they act as important secondary messengers in several plant processes including tolerance to various stresses (Yan et al., 2007). Cd^2+^ can interfere with numerous biochemical and physiological processes including photosynthesis, respiration, nitrogen and protein metabolism, and nutrient uptake (Clemens, 2006; Sanitàdi Toppi & Gabbrielli, 1999) and these results in the increase production of the reactive oxygen species. In lettuce plants under Cd^2+^ stress, the contents of H_2_O_2_, O_2_ and malondialdehyde (MDA) increased while the activities of superoxide dismutase (SOD) and catalase (CAT) declined (Xu et al., 2013). Photosynthesis is one of the most important physiological processes in plants affected by Cd^2+^ toxicity. Higher Cd^2+^ concentrations strongly reduces the maximum photochemical efficiency of PSII, impairs net CO_2_ assimilation rate, decreases nutrient uptake and adversely affects plant growth in lettuce (Dias et al., 2012). The contents of photosynthetic pigments, including chlorophyll a, chlorophyll b and carotenoids decreased under Cd^2+^ stress (Xu et al., 2013). Moreover, Cd^2+^ treatment was found to have increased lipid peroxidation, decreased photosynthesis, shoot growth as well as the contents of nutrient elements (Fe, Mn, Zn and Cu) in lettuce plants. The Cd^2+^ treated plants also accumulated more Cd^2+^ ions in the roots than in the leaves at the end of the experiment (Monteiro et al., 2009). Cd^2+^ is among the major hazardous environmental contaminants and is known as the only heavy metal that poses serious health risks to both humans and animals even at concentrations that are generally not phytotoxic (Ismael et al., 2018). The maximum acceptable limit of Cd^2+^ in leafy vegetables for human consumption is 0.2 mg/kg (FAO/WHO, 2001) and this limit is with reference to the content in the edible part of the crop and not the total amount of Cd^2+^ in the whole plant (Yang et al., 2004). Lettuce is among the high risk crops with regard to Cd^2+^ contamination since the plants can accumulate greater amount of Cd^2+^ without exhibiting marked symptoms of Cd^2+^ toxicity (Zhang et al., 2013).

Plant hormones are not only important for their primary role in regulating plant growth and development (Peto’ et al., 2011), but also for their roles in improving plants tolerance to biotic and abiotic stresses (Krishnamurthy & Rathinasabapathi, 2013). Exogenous application of abscisic acid (ABA) improves plants tolerance to various stresses (Meng et al., 2009). ABA, which is an isoprenoid phytohormone, regulates various physiological processes including stomatal opening and protein storage and provides adaptation to many stresses in plants (Sah, Reddy & Li, 2016). ABA application improves plants tolerance to stress by enhancing the activities of SOD and CAT (Lu et al., 2009). In lettuce plants under drought stress, the application of ABA decreased transpiration, increased biomass production and enhanced the expression of stress marker genes in arbuscular mycorrhizal plants compared with the non-arbuscular mycorrhizal plants (Aroca, Vernieri & Ruiz-Lozano, 2008). The application of ABA also alleviated Cd^2+^ stress in purple flowering stalk (*Brassica campestris* L.) plants by decreasing the contents of MDA and O_2_, increasing the chlorophyll content and enhancing the activities of antioxidant enzymes including SOD, peroxidase (POD), ascorbic peroxidase (APX) and glutathione reductase (GR) (Shen, Niu & Deng, 2017). Contrary to the earlier reports, exogenous ABA caused an increase Cd^2+^ accumulation, decreased biomass and plant height, as well as chlorophyll a, chlorophyll b, total chlorophyll, and carotenoid contents of two Cd^2+^ hyper-accumulator, *Bidens pilosa* ecotypes, when the plants were grown in Cd^2+^-contaminated soil (Liu et al., 2017).

The adverse effects of Cd^2+^ on crop productivity and human health makes it is imperative to develop strategies that will not only improve plants tolerance to Cd but could as well reduce Cd^2+^ levels in the edible parts of crops for food safety. Lettuce (*Lactuca sativa* L.), which is one of the most important leafy vegetables widely grown and consumed in the world (Monteiro et al., 2009; Xu et al., 2013), was used in our current study. The high capacity of lettuce for Cd^2+^ uptake and accumulation makes the plant a potentially high risk crop in terms of Cd^2+^ toxicity in humans (Zhang et al., 2013). Leafy vegetables such as lettuce have been used for toxicological studies due to their high capacity for heavy metal accumulation (Khan et al., 2010). The effect of exogenous ABA application on lettuce under drought stress (Al-Muhairi et al., 2015), *Phaseolus vulgaris* L. and *Sesbania cannabina* under salt stress (Shevyakova et al., 2013; Ren, Kong & Xie, 2018) and pea seedlings under Cd^2+^ stress (Lu et al., 2018) have been reported. Recently, the effect of different concentrations of ABA on growth and Cd^2+^ accumulation in lettuce was reported (Tang et al., 2019). In the current study, however, we investigated the effect of ABA (10 µg L^−1^) on the biochemical, physiological and leaf safety of a Cd^2+^-sensitive lettuce genotype under Cd^2+^ stress. We simultaneously tested the effect of sole ABA and sole ABA-inhibitor (fluridone) to confirm the effect of ABA on the response of lettuce plants under Cd^2+^ treatment.

The present study was conducted based on the hypothesis that exogenous application of ABA can mitigate Cd^2+^-induced stress and increase the food safety of Cd^2+^-sensitive lettuce genotypes. To test the hypothesis, we subjected lettuce seedlings to five treatments (including Control, 100 µM CdCl_2_, CdCl_2_+ABA, 10 µg L^−1^ABA and 0.01 g L^−1^ABA-inhibitor, *fluridone*) and measured lipid peroxidation, antioxidant enzyme activities, photosynthesis, contents of nutrient elements and Cd^2+^ contents, as well as root and leaf biomass. The objectives of our study were: (i) To determine whether exogenous ABA could mitigate Cd^2+^stress in a Cd^2+^-sensitive lettuce genotype and (ii) To determine whether exogenous ABA could improve the food safety of Cd^2+^-sensitive lettuce genotype grown in Cd^2+^ contaminated nutrient solution.

## Materials & Methods

### Plant materials and growth conditions

The study was conducted at the College of Horticulture, Gansu Agricultural University, Lanzhou, Gansu Province, PR China (36°03′N, 103°40′E). The seeds of Cd^2+^-sensitive lettuce cultivar (*Lactuca sativa* L., cv. *Lüsu*), purchased from Gansu Academy of Agricultural Sciences, Lanzhou, China, were used in this experiment. The *Lüsu* genotype was identified as a Cd^2+^-sensitive genotype among other genotypes in our previous studies (Dawuda et al., 2019). The seeds were germinated in a triple-layer filter papers lined in petri dishes within 48 h under light condition in a climate box (temperature 20 °C; light intensity 200 µmol m^−2^ s^−1^ photosynthetic active radiations, PAR; relative humidity 80%). The germinated seeds were sown in trays filled with commercially produced substrate and the seedlings were nursed under greenhouse conditions (average daily temperature 24 ± 2 °C; relative humidity 60–70%; 12 h light) for 14 days. During the first week of growth, the young seedlings were irrigated with 1/4-strength Hoagland’s nutrient solution (Hoagland & Arnon, 1950) with pH 5.5. After the first week, watering was done using $ \frac{1}{2} $ strength of the Hoagland’s nutrient solution until the seedlings were 28 days old.

The 28 days old seedlings were transferred to Hoagland’s nutrient solution and allowed for four days to acclimate in a climate controlled room (25 °C/22 °C light/dark periods, 75% relative humidity and 14 h light period with 200 µmol m^−2^ s^−1^ PAR) before treatment application. In all, 288 seedlings were grown in 72 opaque hydroponic containers (1.5 L capacity each). Each of the containers was filled with 1.2 L of full-strength Hoagland’s nutrient solution and supplied with 4 seedlings. The nutrient solution was changed at 4 days interval until the plants were harvested for data collection.

### Experimental design and treatments

The experiment was conducted in a climate box using 28 days old seedlings. Five treatments (Table 1), including (i) Control, (ii) 100 µM Cd (CdCl_2_), (iii) 10 µg L^−1^ ABA, (iv) 0.01 g L^−1^ ABA-inhibitor (fluridone) and (v) CdCl_2_+ABA were applied in a completely randomized design with 3 replications. We used the 100 µM CdCl_2_ based on our previous experiment in which we evaluated the response of four lettuce genotypes to Cd^2+^ stress (Dawuda et al., 2019). The concentration of ABA used in this experiment was chosen based on previous experiment (Al-Muhairi et al., 2015; Al-Muhairi et al., 2016). The concentration of fluridone (C_19_H_14_F_3_NO, 99.4%, Sigma-Aldrich, USA), which is ABA-inhibitor, was also chosen based on previous studies conducted by Vysotskaya and co-authors (Vysotskaya et al., 2018). The 100 µM CdCl_2_ treatment was supplied through the hydroponic nutrient solution while the ABA and fluridone treatments were sprayed on either side of the leaves. Each group of four plants per pot was sprayed uniformly with 10 ml (approximately 2.5 ml per plant) of either ABA or fluridone depending on the treatment. The CdCl_2_ treated plants and the control plants were sprayed with distilled water in a similar manner as the ABA and fluridone treatment. The plants were sprayed for four days at 24 h intervals and then monitored for a further 10 days before the experiment was ended.

**Table 1:** Summary description of the treatments applied in the study.

Treatment	CdCl_2_ (100 µM)	Fluridone (0.01 g L^−1^)	ABA (10 µg L^−1^)
*Control	**−**	**−**	**−**
*CdCl_2_	**+**	**−**	**−**
CdCl_2_+ABA	**+**	**−**	**+**
ABA	**−**	**−**	**+**
Fluridone	**−**	**+**	**−**

**Notes.**

Chemical not applied (-); Chemical applied (+); Plants sprayed with distilled water in a similar manner as the ABA and fluridone application (*).

Each experimental unit consisted of three plastic containers (1.5 L capacity each). Each container was supplied with 4 plants and a total of 36 plants were used per treatment. The control plants were grown in normal Hoagland’s nutrient solution. The ABA, fluridone and distilled water were applied using separate 100 mL capacity hand sprayer. The treatments were applied to the 28 days old seedlings. The first spraying of plants with ABA, fluridone or distilled water was done just before the plants were exposed to 100 µM CdCl_2_ treatment for the first time. Leaf and root samples were collected at 10 days after start of treatments for the determination of lipid peroxidation, antioxidant enzymes activities and photosynthesis indexes. Data on the contents of nutrient elements and cadmium, as well as the biomass of the plants were measured at 14 days after treatments.

### Data measurements

Root and leaf samples were collected from four plants which were randomly selected per replicate at 10 days after the start of treatment application for the determination of biochemical parameters including lipid peroxidation and antioxidant enzymes activities. The contents of photosynthetic pigments, gas exchange parameters and relative water content of leaves were also measured at 10 days after the start of treatments. The contents of nutrient elements and Cd^2+^, as well as the root and leaf biomass of the plants were measured at the end of the experiment (14 days after start of treatments) using six plants per replicate.

### Determination of hydrogen peroxide (H_2_O_2_) and malondialdehyde (MDA) contents

Contents of H_2_O_2_ in the roots and leaves samples were determined following the procedure described by Junglee et al. (2014) with slight modifications. Briefly, 0.1 g fresh samples were ground with liquid nitrogen in mortar with pestle and the homogenate was transferred into two mL capacity centrifuge tubes which were then kept in ice bath. We added 1.5 mL 0.1% trichloroacetic acid (TCA) and the homogenate were centrifuged at 12,000 g for 15 min under 4 °C. Then, 0.5 ml of the supernatant was thoroughly mixed with 0.5 mL PBS (pH 7.0) and one mL 1 mol L^−1^ KI. The mixture was placed under constant temperature (28 °C) for 1 h. The absorbance was measured at 390 nm and the contents of H_2_O_2_ were calculated using the H_2_O_2_ reference standard curve (0, 1, 2, 3, 4 and five mmol L^−1^).

Contents of MDA in leaf and root samples were measured as described by Fazeli, Ghorbanli & Niknam (2007) with some modifications. In brief, the fresh leaf and root tissues (0.3 g each) were ground separately in ice bath with two mL 0.05 mol L^−1^ TCA. The homogenate was centrifuged at 10,000 g for 5 min at 25 °C and then we added five mL 0.5% (m/v) thiobarbituric acid (TBA) solution to the supernatant. The extract was placed in a hot water bath for 10 min, and then quickly moved to cold water bath. After cooling, the solution was centrifuged again at 10,000 g for 10 min at 25 °C. The absorbance of the supernatant was measured at 532 nm; and the value for nonspecific absorption at 600 nm was subtracted. The content of MDA was calculated from the molar extinction coefficient of MDA-TBA at 155 mmol^−1^ cm^−1^.

### Determination of antioxidant enzymes activities

Root and leaf samples were collected at seven days after treatments application and stored at −80 °C for enzymes assays. For each enzyme, about 0.5 g of the root tissue was ground in liquid nitrogen with a mortar and pestle and then homogenized in 5 ml of 0.1 M phosphate buffer (pH 7.5), containing 0.5 mM ethylene-diamine-tetra-acetic acid (EDTA). Each homogenate was centrifuged at 12,000× g for 15 min at 4 °C, and the supernatant was aliquot for superoxide dismutase (SOD, EC 1.15.1.1), catalase (CAT, EC 1.11.1.6) and peroxidase (POD, EC 1.11.1.7) activity assays. The supernatants were used for the determination of enzymatic activity. The SOD, POD and CAT activities were measured as described by Giannopolitis & Ries (1977), Chance & Maehly (1955) and Nakano & Asada (1981), respectively.

### Determination of contents of chlorophyll and carotenoids

In brief, leaf tissues were collected from six plants per treatment at 10 days after the start of treatments and 0.1 g (fresh weight) was ground in 10 ml 85% acetone. The absorbance or optical density (OD) values for the extracts were measured at 663, 645 and 440 nm after 48 h using a spectrophotometer (UV 1800, Tokyo, Japan) and the contents of chlorophyll a, chlorophyll b, chlorophyll a+b and carotenoids were measured following the method of Zhang (1992).

### Determination of gas exchange parameters

Net photosynthetic rate (P_N_), stomatal conductance (Gs), transpiration rate (Tr) and intercellular concentration of CO_2_(Ci) were determined using a portable photosynthetic system (CIRAS-2, PP System, UK) following the method of Gu et al. (2007) with slight modification. In brief, two fully expanded leaves from the top of two plants per treatment were selected for the measurement. The photosynthetic photon flux density (20000 lux), ambient CO_2_concentration (380 µmol mol^−1^), leaf temperature (22 °C) and relative humidity (65–75%) were maintained in the climate box for period of the measurements.

### Determination of relative water content

The relative water content (RWC) was measured as described by Hayat et al. (2007). In brief, fully expanded leaves were collected from each treatment and four fresh leaf discs (two cm diameter, excluding midrib) were taken, weighed and immediately floated on deionized water in petri dishes. Each petri dish was supplied with 100 ml deionized water to saturate the leaf disc for 24 h, under dark condition. At 24 h, the leaf discs were removed from the water and blotted with tissue papers to remove the adhering water and the turgor mass recorded. The leaf discs were then dried at 70° C for 48 h. The RWC was calculated as follows:

\begin{eqnarray*}\mathrm{RWC}= \frac{\mathrm{FW}-\mathrm{DW}}{ \mathrm{TW}-\mathrm{DW}}  \mathrm{X} 100 \end{eqnarray*}

where, FW is fresh leaf weigh; DW is dry leaf weight; TW is turgor weight of leaf.

### Determination of contents of nutrient elements and cadmium

The nutrients and Cd^2+^ contents were measured according to the method of Azevedo et al. (2005) with some modifications. In briefly, leaf and root samples were collected from three lettuce plants for each treatment and the fresh weights determined. The samples were washed several times in deionized water, put in envelopes and dried at 80 °C until the dry weight remained constant. The dried samples were ground and sieved with 0.25 mm mesh. The fine samples (1.0 g each) were put into crucibles and burnt to ash at 550 °C for 5–6 h. The ash samples were allowed to cool and one mL nitric acid solution was added and mixed thoroughly. The mixture was then filtered and the volume made up to 50 mL by adding drops of distilled water. The concentrations of the elements were determined using the atomic absorption spectrophotometer with the OD set as follows: 422.6 nm (Ca); 285.2 nm (Mg); 324.8 nm (Cu); 248.3 nm (Fe); 213.8 nm (Zn); 228.8 nm (Cd). The contents of the nutrient elements (leaf) and cadmium (leaf and root) were calculated using the following formula:

*ω* = ((c-c_0_) ×50 ×N ×1000) / (m ×D)

where:

*ω*: Content of nutrient element

c: Concentration of elements in sample solution (mg/L)

c_0_: Concentration of elements in blank control solution (mg/L)

N: Dilution multiple

m: Weight of sample (g)

D: Is a constant, when the value is represented by mg/kg, D is 10^3^; when the value is represented by g/kg, D is 10^6^.

50: The volume of the sample solution.

### Plant biomass and biomass response to stress (BRS)

The fresh leaf and root biomass per plant were measured immediately after harvest at 14 days after the start of the treatments. The leaf and root samples were put in separate envelops and dried at 65 °C for 48 h for the dry weights. The biomass response to stress (BRS) which gives an indication of the tolerance of the plant or plant organ to stress was calculated as described by Wilkins (1978) with slight modification. The BRS was calculated using the average plant weight of each treatment at 14 days after start of treatments as follows:

\begin{eqnarray*}\mathrm{BRS}= \frac{\text{Plant in stress}}{\text{Plant without stress}} x100. \end{eqnarray*}

### Data analyses

The data measured were subjected to a one-way analysis of variance using Genstat statistical software (12th edition, Lawes Agricultural Trust, Rothamsted Experimental Station, UK). The treatment means were separated by the least significance difference (LSD) test at 5%. The results are presented as means + standard deviations. Different letters assigned to the bars indicate significant differences among the treatments by LSD (*P* < 0.05). Graphs were prepared using Graphpad Prism version 8.0.

## Results

### Hydrogen peroxide (H_2_O_2_) and malondialdehyde (MDA) contents

**Figure 1:**
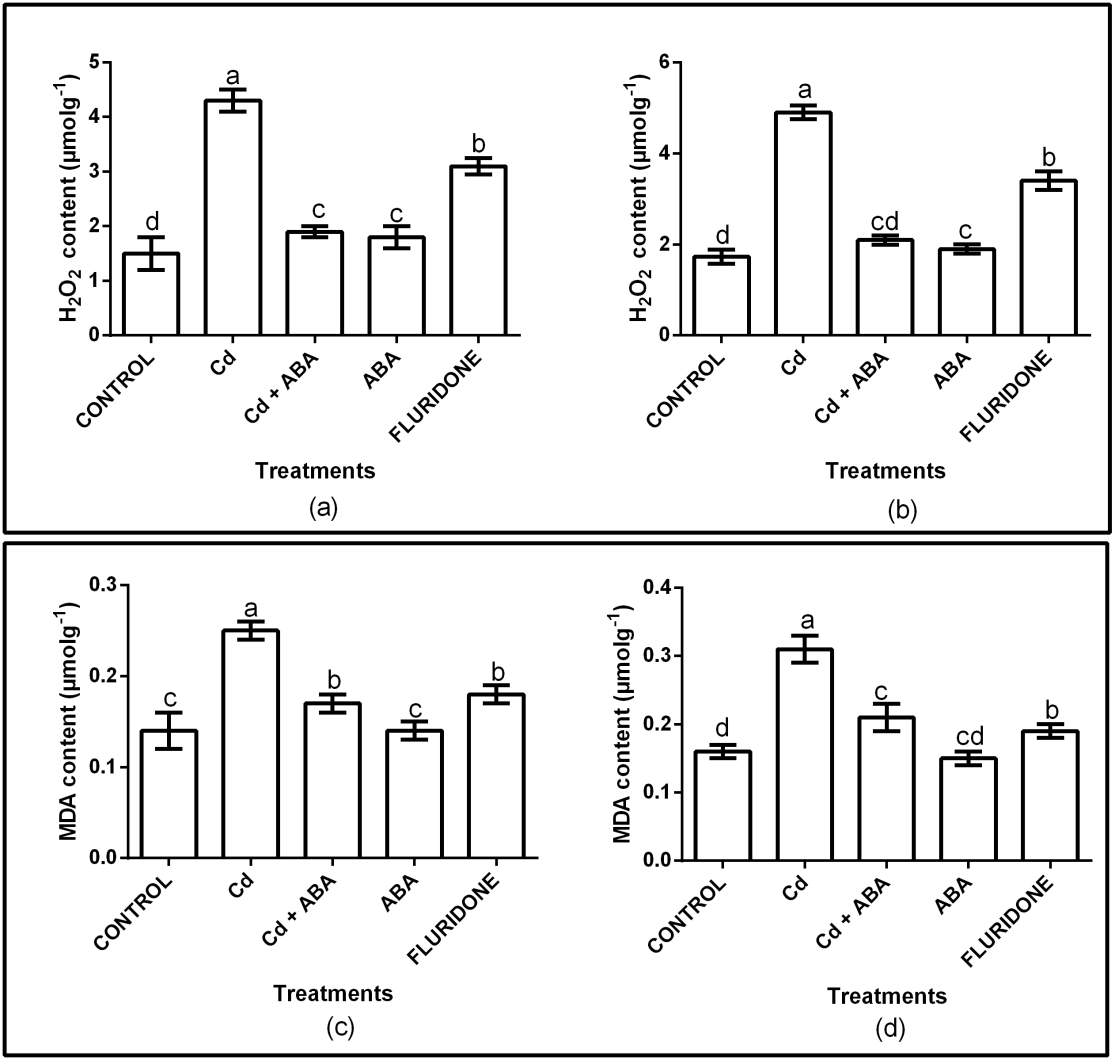
Effect of treatments on contents of H_2_O_2_ (A, leaf; B, root) and MDA (C, leaf; D, root) of lettuce plants. Data shown are treatment means ± SD of three replications. Bars assigned with different lower case letters indicate significant differences by LSD test (*P* < 0.05). Hydrogen peroxide (H_2_O_2_); Malondialdehyde (*MDA*), measured at 10 days after start of treatments.

The effect of CdCl_2_ on oxidative stress was determined by measuring the contents of H_2_O_2_ and MDA in the root and leaf tissues of the plants. The H_2_O_2_ and MDA contents in both roots and leaves were significantly (*P* < 0.001) affected (Fig. 1A). Relative to control plants, the CdCl_2_ treatment increased the contents of H_2_O_2_ in the roots and leaves by 65.3% and 65.1%, respectively. Moreover, CdCl_2_ treatment increased the contents of MDA in the roots and leaves by 48.4% and 44%, respectively (Fig. 1B). However, application of ABA to the plants under CdCl_2_ treatment (CdCl_2_+ABA) suppressed the accumulation of H_2_O_2_ in both roots and leaves by about 3.4 and 3.1 folds, respectively. In addition, exogenous ABA inhibited the accumulation of MDA in the roots and leaves of the CdCl_2_ treated plants, decreasing it by 2 and 2.5 fold, respectively. The application of ABA to unstressed lettuce plants slightly increased the H_2_O_2_ contents in the roots (10.5%) and leaves (5.6%). However, the MDA contents in the roots and leaves of the sole ABA treated plants were similar to the control plants. Moreover, the fluridone treatment also increased the contents of H_2_O_2_ in the roots by 50% and in leaves by 51.6%. Similarly, the contents of MDA increased in the roots and leaves of the fluridone treated plants by 15.8% and 22%, respectively.

### Antioxidant enzymes activities

**Figure 2:**
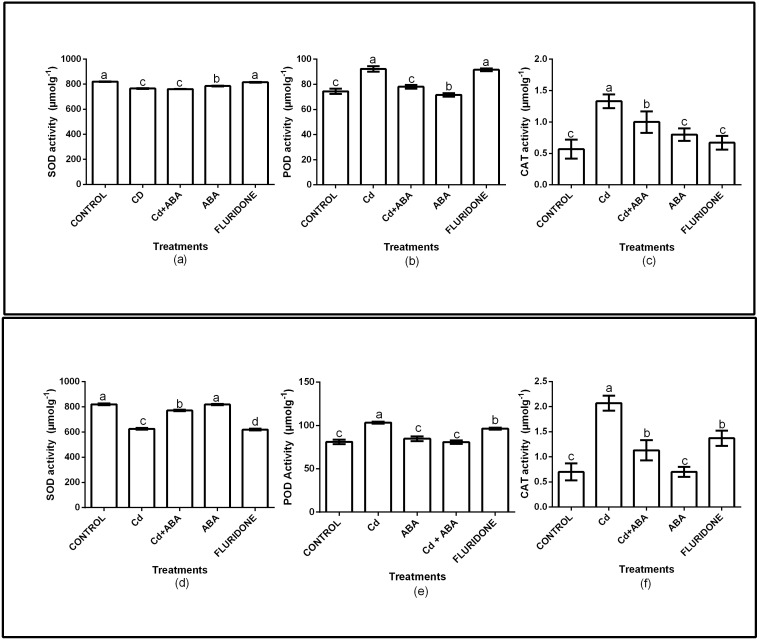
Effect of treatments on antioxidant enzymes (SOD, POD, CAT) activities in leaves (A–C) and roots (D–F) of lettuce plants. Data shown are treatment means ± SD of three replications. Bars assigned with different lower case letters indicate significant differences by LSD test (*P* < 0.05). Superoxide dismutase (*SOD*); Peroxidase (*POD*) and Catalase (*CAT*), measured at 10 days after start of treatments.

The role of exogenous ABA in the antioxidant enzymes protection of the lettuce plants was determined by measuring the SOD, CAT and POD activities in the leaves and roots of the plants (Fig. 2). Relative to the control plants, the CdCl_2_ treatment significantly (*P* < 0.001) decreased SOD activities in both leaves and roots by 6.7% and 23.7%, respectively. However, the application of ABA to the plants under CdCl_2_ treatment resulted in marginal increases in the leaf (4.3%) and root (5.9%) SOD activities. The ABA treated plants had leaf and root SOD activities similar to the control plants. The fluridone treatment also decreased the SOD activities of the leaves and roots of the plants by 7.2% and 24.4%, respectively. The results also showed that the CAT and POD activities of the plants under CdCl_2_ stress increased (Fig. 2). The CAT activities in the leaf and root of CdCl_2_treated plants increased by 57.1% and 66.2% respectively. The leaf and root POD activities of the CdCl_2_treated plants also increased by 19.2% and 21.6%, respectively. However, the leaf and root POD activities of CdCl_2_+ABA treated plants were 4.6% and 4.3%, respectively. The CAT and POD activities of the sole ABA treated plants were similar to those of the control plants. The results also showed that the CAT and POD activities of the fluridone treated plants were high and statistically similar to those of the CdCl_2_ treatment.

### Chlorophyll (Chl) and carotenoids contents

**Figure 3:**
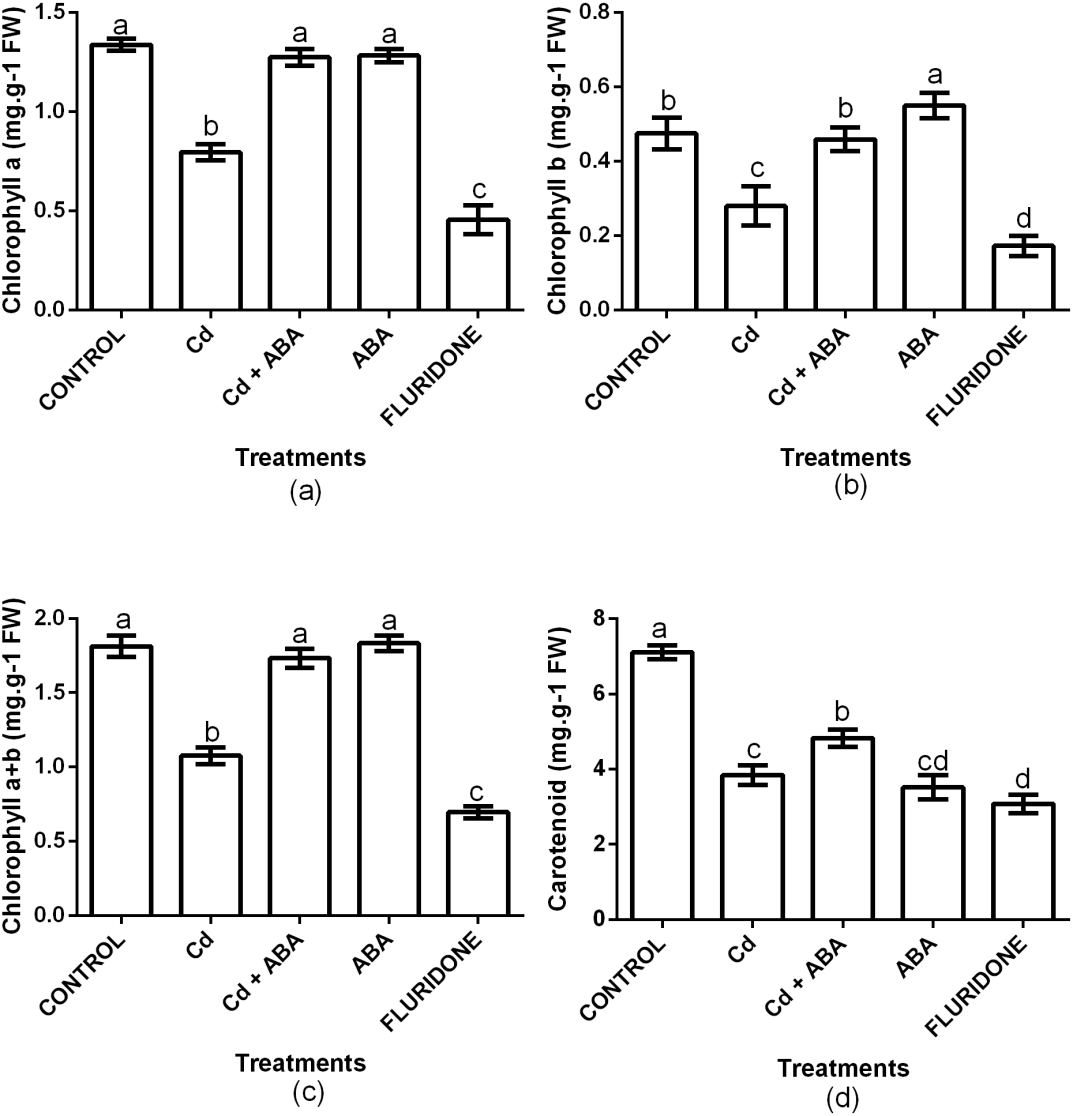
Effect of treatments on contents of chlorophyll a (A), chlorophyll b (B), chlorophyll a+b (C) and carotenoids (D) in lettuce plants measured at 10 days after start of treatments. Data shown are treatment means ± SD of three replications. Bars assigned with different lower case letters indicate significant differences by LSD test (*P* < 0.05).

The contents of Chl a, Chl b, Chl a+b and carotenoids were significantly (*P* < 0.001) affected by the treatments (Fig. 3). Relative to the control plants, CdCl_2_ stress decreased the contents of chlorophyll a, chlorophyll b and chlorophyll a+b by 40.3%, 41.1% and 40.7%, respectively. Moreover, CdCl_2_ treatment decreased the content of carotenoids by 45.8%. However, the application of ABA to the lettuce plants under Cd^2+^ stress maintained the contents of chlorophyll and carotenoids at levels that were similar to the control plants. The decreases in the contents of Chl a, Chl b, Chl a+b and carotenoids in the Cd+ABA treated plants were only 4.2%, 3.7%, 4.4% and 32.1%, respectively. The contents of Chl a, Chl b and Chl a+b in the sole ABA treated plants were statistically similar to those of the control plants. However, the application of ABA to unstressed plants caused a 50.5% decrease in the content of carotenoids, relative to the control plants. The application of fluridone also decreased the contents of Chl a, Chl b, Chl a+b and carotenoids by 66%, 63.8%, 61.6% and 56.7%, respectively.

### Gas exchange parameters

**Figure 4:**
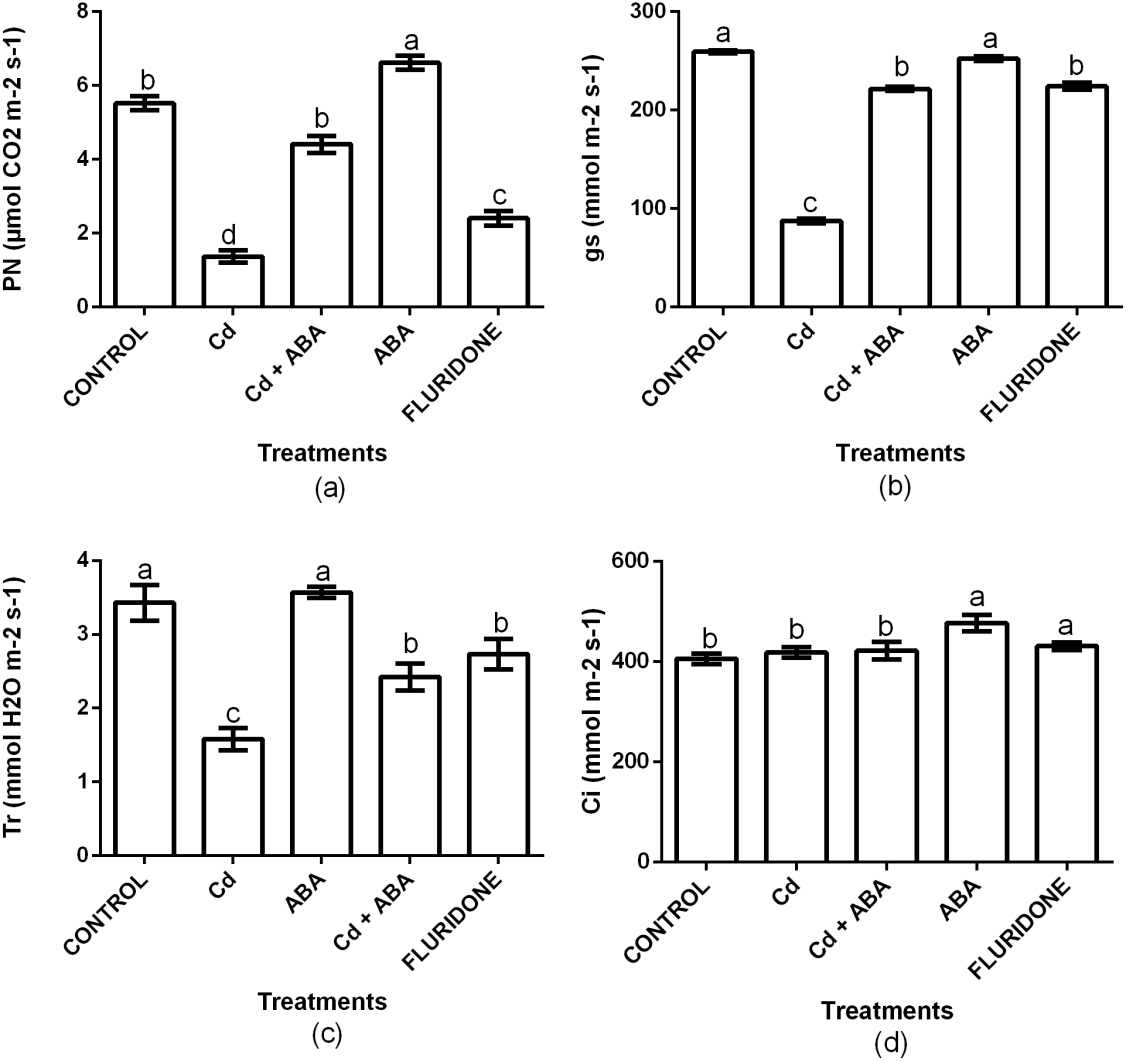
Effect of treatments on P_N_ (A), Gs (B), Tr (C) and Ci (D) of lettuce plants measured at 10 days after start of treatments. Data shown are treatment means ± SD of three replications. Bars assigned with different lower case letters indicate significant differences by LSD test (*P* < 0.05). Net photosynthesis (P_N_); Stomata conductance (Gs); Transpiration rate (Tr); Intercellular CO_2_ (Ci).

To investigate the role of ABA in regulating photosynthesis in lettuce plants under CdCl_2_ stress, we measured net photosynthesis (P_N_), stomata conductance (Gs), transpiration rate (Tr) and intercellular CO_2_ (Ci). Relative to the control plants, CdCl_2_ significantly (*P* < 0.001) decreased the P_N_, Gs and Tr by 75.3%, 66.3% and 53.8%, respectively (Fig. 4). However, the Ci was statistically the same among the control, the CdCl_2_, CdCl_2_+ABA and fluridone treated plants. The adverse effects CdCl_2_ on the gas exchange indexes was reduced by the application of ABA. Relative to the control plants, the decrease in P_N_, gs and Tr in the CdCl_2_+ABA treated plants were 20.1%, 14.5% and 29.3%, and these were lesser than those caused by the sole CdCl_2_ treatment. Moreover, the CdCl_2_+ABA treated seedlings had 3.8% higher Ci compared with the control plants. The application of ABA to unstressed lettuce plants caused 16.6% and 15% increases in P _N_ and Ci respectively, over the control plants. However, the Gs and Tr of the CdCl_2_+ABA treated plants and the control plants were statistically the same. The application of fluridone also decreased P_N_, Gs and Tr by 56.5%, 13.5% and 20.3% respectively. However, the Ci of the fluridone treated plants and the control plants were statistically the same.

### Relative water content (RWC) of leaf

**Figure 5:**
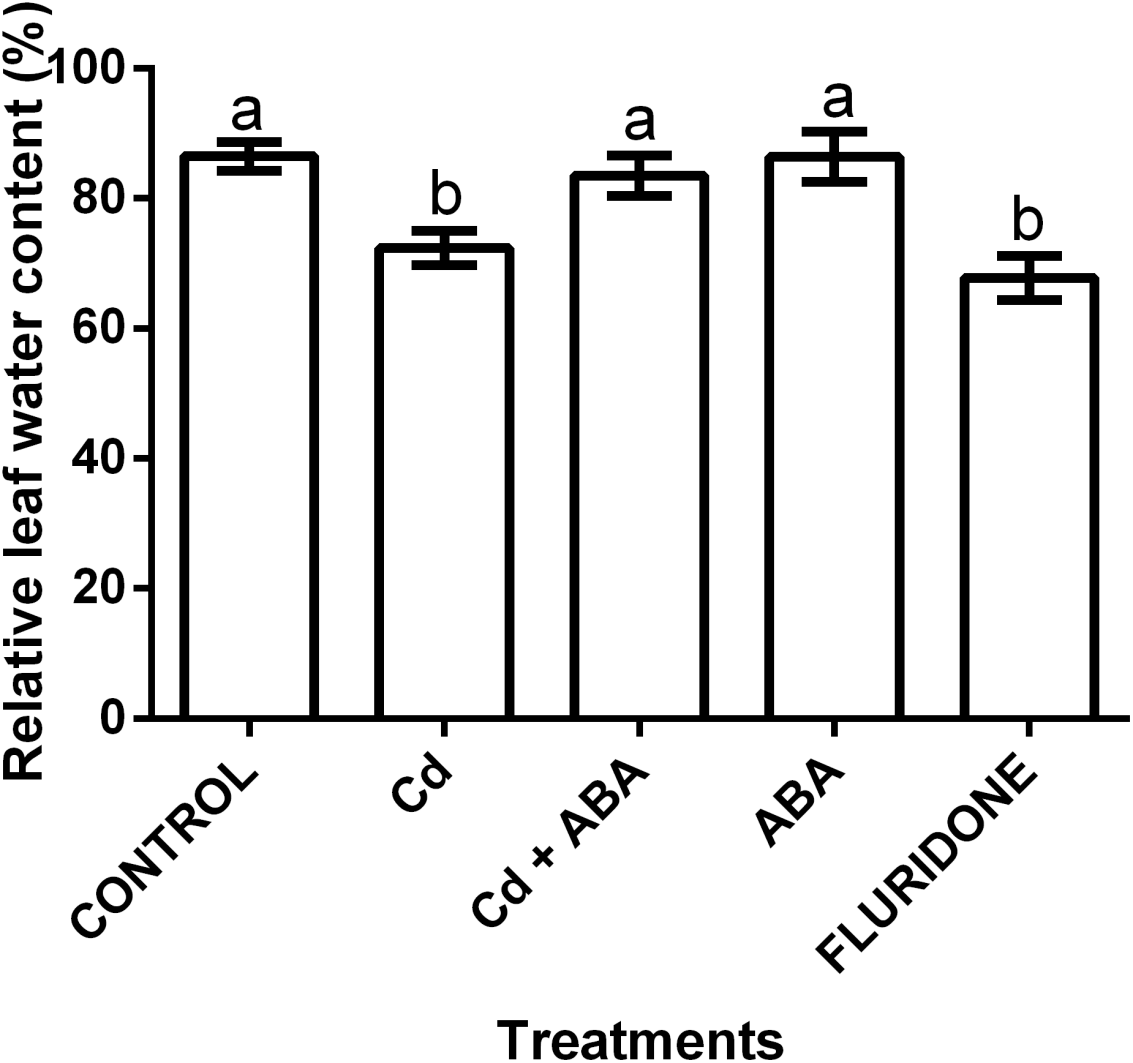
Effect of treatments on relative water contents (%) of lettuce plants measured at 14 days after start of treatments. Data shown are treatment means ± SD of 3 replications. Bars assigned with different lower case letters indicate significant differences by LSD test (*P* < 0.05).

The application of the treatments significantly (*P* < 0.001) affected the relative water content (RWC) of the lettuce plants (Fig. 5). The control seedlings, the ABA and the CdCl_2_+ABA treated seedlings had greater leaf RWCs of 86.5%, 86.4% and 83.5%, respectively. The CdCl_2_ and fluridone treated plants had the least leaf RWCs of 72.4% and 67.8%, respectively.

### Macro and micro nutrient elements

The results on macro and micro nutrient elements are presented in Table 2. At 14 days after treatment application, the contents of the nutrient elements (except Cu) were significantly (*P* < 0.001) affected. Generally, the CdCl_2_ and fluridone treatments greatly decreased the contents of the nutrient elements when compared with the control plants. The CdCl_2_ treatment decreased the contents of Mg, Ca, Zn, Fe and Mn by 41.3%, 31.6%, 54.5% 29.4% and 68.9%, respectively. In most cases, the application of ABA to the unstressed plants maintained the contents of the nutrients elements to levels similar to the control plants. Although the contents of nutrient elements in the Cd+ABA treated plants were in some cases lower than those of the control plants, they were generally higher than those of the sole CdCl_2_ treatment. The application of ABA to unstressed plants maintained the contents of the nutrient elements (except Mn) in the leaves to levels similar to the control plants. With the exception of Cu, fluridone treatment also decreased the contents of nutrient elements to levels lower than the control plants.

**Table 2:** Contents of nutrient elements (mg g^−1^ DW) in the leaves of the lettuce plants measured at 14 days after start of treatments.

Treatment	Mg	Ca	Zn	Fe	Cu	Mn
Control	4.6+0.2a	7.6+0.20a	0.22+0.02a	0.17+0.01a	0.007+0.006a	0.45+0.03a
Cd	2.7+0.1d	5.2+0.15d	0.10+0.02b	0.12+0.01b	0.003+0.001a	0.14+0.01c
Cd+ABA	3.7+0.1b	6.5+0.12b	0.19+0.02a	0.17+0.01a	0.006+0.005a	0.40+0.02b
ABA	4.5+0.2a	7.6+0.10a	0.22+0.01a	0.17+0.01a	0.006+0.006a	0.41+0.03b
Fluridone	3.1+0.2c	6.2+0.12c	0.11+0.02b	0.12+0.01b	0.002+0.001a	0.17+0.02c
LSD (0.05)	0.29	0.26	0.03	0.016	NS	0.038
CV (%)	4.3	2.1	10.6	6.0	28.4	6.8

**Notes.**

Data shown in Table 2 are treatment means ± SD of 3 replications. Means assigned with different lower case letters in the same column indicate significant differences by LSD test (*P* < 0.05). NS, denotes no significant difference among the treatments.

### Cadmium contents

**Figure 6:**
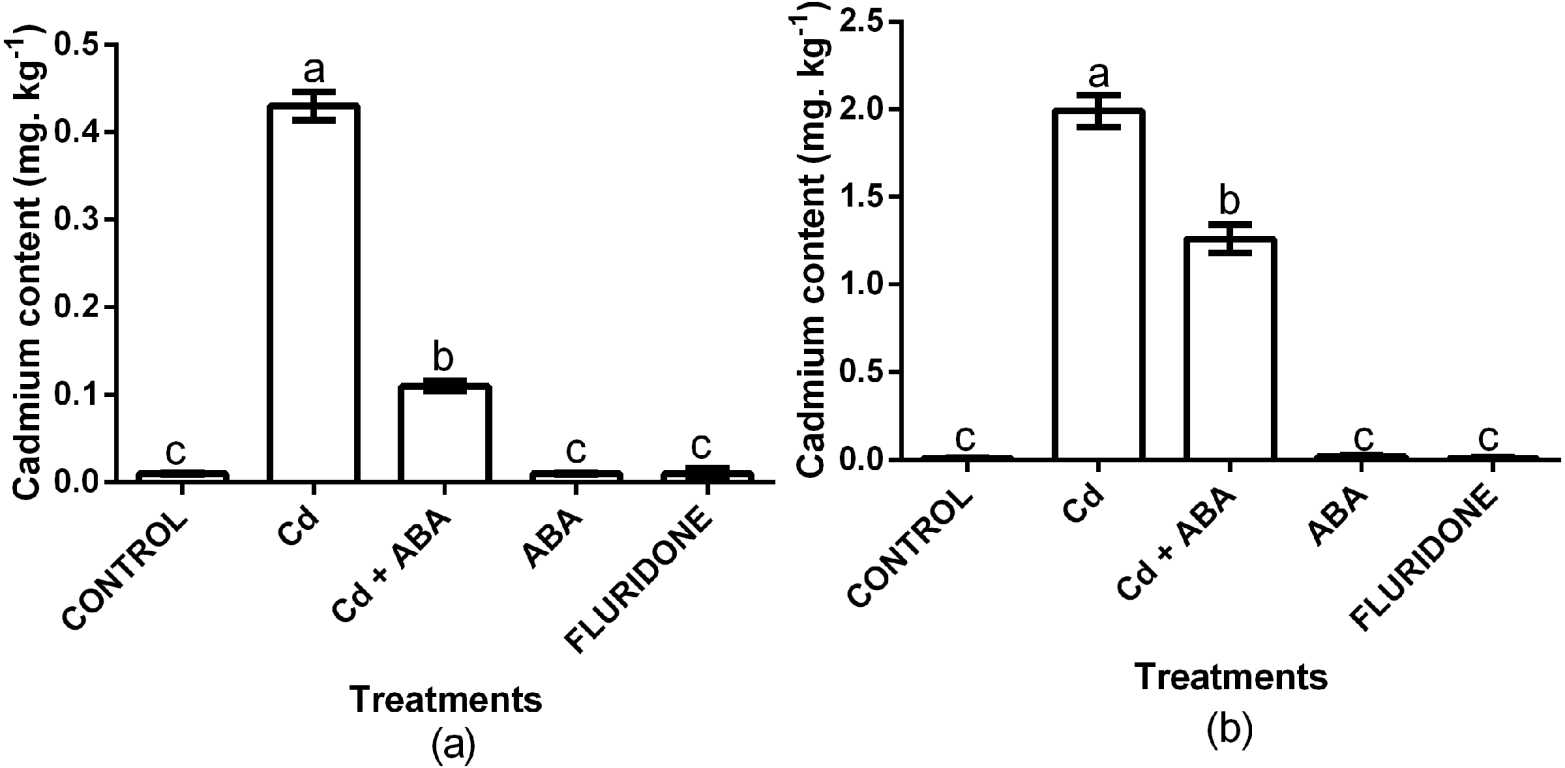
Effect of treatments on contents of cadmium in leaves (A) and roots (B) of lettuce plants measured at 14 days after start of treatments. Data shown are treatment means ± SD of 3 replications. Bars assigned with different lower case letters indicate significant differences by LSD test (*P* < 0.05).

We analyzed the contents of Cd^2+^ in the root and leaf samples from the various treatments (Fig. 6). In comparison with the control plants, the 100 µM CdCl_2_ treated plants had 97.7% and 99.5% more Cd^2+^ in the leaf and root of the plants, respectively. Even though the CdCl_2_+ABA treated plants also had higher leaf and root Cd^2+^ contents, the increases were relatively lower compared with the sole CdCl_2_ treatment. Moreover, the content of Cd^2+^ in the leaves of the CdCl_2_+ABA treated plants (0.11 mg. kg^−1^ FW) was lower than the 0.2 mg kg^−1^ recommended for leafy vegetables.

### Biomass, biomass response to stress (BRS) and morphology of lettuce plants

**Figure 7:**
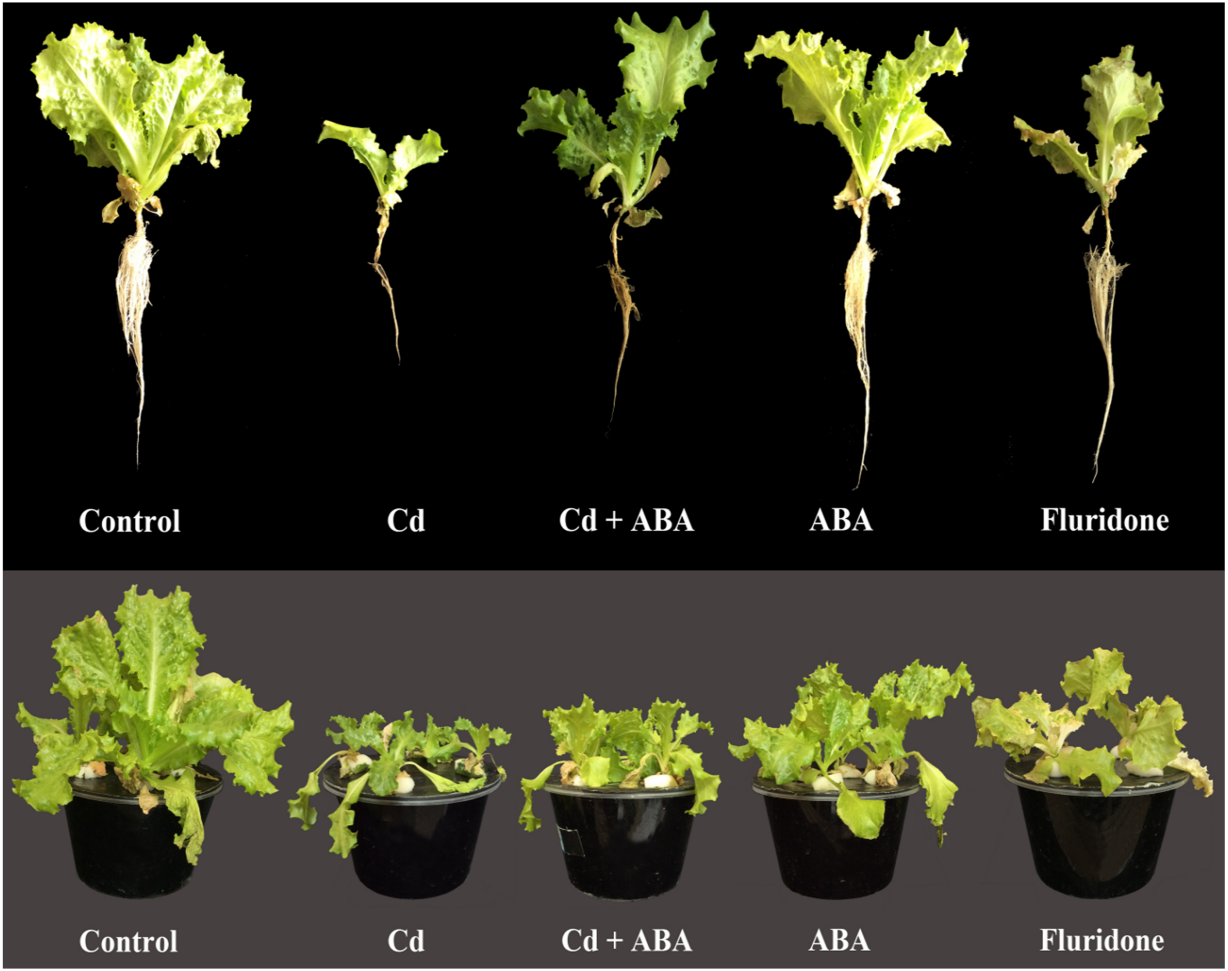
Effect of treatments on the morphology of lettuce plants at 14 days after the start of treatments. *Control* (Plants grown in normal nutrient solution); *Cd* (Plants grown in nutrient solution supplied with 100 μ M CdCl_2_); *Cd+ABA* (P). The control and 100 μ M CdCl_2_ treated plants were also sprayed with distilled water in a similar manner as the application of ABA and fluridone.

Relative to the control plants, the 100 µM CdCl_2_ treatment significantly (*P* < 0.001) decreased fresh leaf weight (FLW), fresh root weight (FRW), dry leaf weight (DLW) and dry root weight (DRW) of the plants by 59.9%, 74.6%, 73% and 66.7%, respectively (Table 3). In addition, the biomass response to Cd stress (BRS) of the CdCl_2_ treated plants was the least (36.4%). However, the Cd+ABA treated plants had higher BRS (81.2%) compared to the sole CdCl_2_ treated plants. Moreover, the Cd+ABA treated plants suffered lesser reductions in FLW (14.9%), FRW (31.1%), DLW (31.2%) and DRW (33.3%) compared with the sole CdCl_2_ treated plants. The root and leaf biomass of plants treated with sole ABA were relatively similar to those of the control plants. However, the application of ABA-inhibitor (fluridone), also decreased the root and leaf biomass of the lettuce plants. Furthermore, the CdCl_2_ and fluridone treated plants had the least BRS (36.4% and 58.3%, respectively) indicating the plants suffered greater stress from Cd^2+^ and the ABA-inhibitor (Table 2). In comparison with the CdCl_2_ treated plants, the Cd+ABA treated plants had higher BRS (%) value of 81.2%, indicating that ABA improved the tolerance of the plants to the Cd-induced stress. Figure 7 shows the appearance of the plants at 14 days after start of treatments. The control and ABA treated plants appeared similar in terms of size. However, the leaves and roots of the sole CdCl_2_ treated plants were greatly reduced in size. In addition, the root system appeared relatively brownish in color. Even though the CdCl_2_+ABA treated plants were not as large as the control and the sole ABA treated plants, they were relatively larger than the sole CdCl_2_ treated plants. Although fluridone (ABA-inhibitor) treatment did not decrease the leaf and root sizes of the plants as much as the CdCl_2_, the roots were relatively brownish in color and portions of the leaves appeared chlorotic and bleached.

**Table 3:** Effect of treatments on root and leaf biomass (g plant^−1^) and plant biomass response to stress (BRS) at 14 days after start of treatments.

Treatment	Root (g plant^−1^)		Leaf (g plant^−1^)		BRS (%)
	Fresh	Dry	Fresh	Dry	
Control	0.784+0.080a	0.027+0.011a	2.420+0.010a	0.168+0.015a	100
CdCl_2_	0.199+0.012d	0.009+0.005b	0.966+0.0658d	0.071+0.010d	36.4
CdCl_2_+ABA	0.540+0.040b	0.018+0.001ab	2.063+0.032b	0.149+0.005b	81.2
ABA	0.732+0.025a	0.021+0.009ab	2.410+0.030a	0.166+0.032a	98.1
Fluridone	0.409+0.015c	0.017+0.005ab	1.460+0.319c	0.116+0.011c	58.3
LSD (0.05)	0.07	0.01	0.23	0.03	
CV (%)	9.1	38.5	7.9	9.1	

**Notes.**

BRS was not subjected to analysis of variance.

## Discussion

Cadmium-induced stress in plants increases lipid peroxidation, which is characterized by increased accumulation of reactive oxygen species such as hydrogen peroxide (H_2_O_2_), superoxide anion (O_2_), singlet oxygen (^1^O_2_) and hydroxyl radical (-OH) in plant cells (Tripathi et al., 2017; Sarvikas & Tyystjärvi, 2010). Reactive oxygen species (ROS) are harmful to plant cells at higher concentrations but in the absence of stress, they act as important secondary messengers in several plant processes including tolerance to various stresses (Yan et al., 2007). Excessive production of ROS is harmful to cells mainly because of their reactions with lipids, proteins, and nucleic acids which results in lipid peroxidation, membrane leakage, enzyme inactivation, and DNA break down or mutations (Halliwell & Gutteridge, 2007). Moreover, the exposure of plants to various kinds of stresses results in increased accumulation of MDA, which is the end product of oxidative stress (Monteiro et al., 2009). In the current study, the 100 µM CdCl_2_ treatment caused oxidative stress by increasing the production of H_2_O _2_ andMDA in the roots and leaves of the lettuce plants (Fig. 1). In addition, the 100 µM CdCl_2_ decreased the SOD activity of the plants whiles the CAT and POD activities in the roots and leaves of the plants increased (Fig. 2). In previous studies involving lettuce, Cd^2+^ stress increased the contents of H_2_O_2_ (Xu et al., 2013) and MDA (Loi et al., 2018). A recent study also showed that Cd^2+^ stress decreased SOD activity but CAT and POD activities increased in the roots of four lettuce genotypes (Dawuda et al., 2019). However, the application of ABA to the seedlings under Cd^2+^ treatment mitigated the oxidative stress by inhibiting the excess production of H_2_O_2_ and MDA (Fig. 1). In addition, the application of ABA also maintained the activities of antioxidant enzymes at levels lower than the sole CdCl_2_ treatment (Fig. 2). ABA is reported to act in several ways to improve the antioxidant defense system in plant cells (Peleg & Blumwald, 2011). Exogenous ABA application alleviated Cd ^2+^ stress in *Brassica campestris* (Shen, Niu & Deng, 2017) whiles pretreatment of rice seeds with ABA protected the plants against salt stress (Sripinyowanich et al., 2013).

Heavy metal stress decreases the contents of chlorophyll in plants by interfering with the activities of the biosynthetic enzymes, particularly, protochlorophyll oxydoreductase and chlorophyll synthase (Loi et al., 2018). Cd^2+^ interferes with chlorophyll biosynthesis by competing for space with Mg^2+^ in the chlorophyll molecule (Titov, Talanova & Kaznina, 2011). In our current study, Cd^2+^ stress decreased the contents of Chl a, Chl b and Chl a+b of the plants relative to the control plants. Our results was similar to the findings of Dias et al. (2012) who found that Cd^2+^ stress decreased the contents of Chl (a and b) in lettuce plants but the effect was more on chlorophyll a than chlorophyll b. In another experiment involving strawberry, increasing Cd^2+^ concentration from 0 to 60 mg kg−1 soil decreased the contents of both chlorophyll a and chlorophyll b but chlorophyll a was more affected (Muradoglu et al., 2015). However, our experiment showed that Cd^2+^ stress decreased the contents of chlorophyll a (40%) and b (41%) almost equally in the lettuce plants (Fig. 3). The chlorophyll contents in several other plant species were greatly reduced when the plants were exposed to Cd^2+^ (Lo’pez-Milla’n et al., 2009; Gill, Khan & Tuteja, 2012). In the current study, the 100 µM CdCl_2_ treatment also decreased the content of carotenoid in the seedlings. This result is in contrast with the findings of Dias et al. (2012) who reported that the contents of carotenoids in lettuce plants exposed to 10 µM Cd and 50 µM Cd increased. The differences in these results could be due to differences in the Cd concentrations applied and/or due to genotypic variations. Even though the contents of carotenoids in the sole ABA and Cd+ABA treated plants were relatively lower than the control, these treatments could maintain the contents of chlorophyll a, b and a+b, to levels similar to the control plants. In a study involving two tomato genotypes, the application of different concentrations of ABA increased the contents of chlorophyll and carotenoids under normal growth conditions (Barickman, Kopsell & Sams, 2014). In another experiment, seed treatment with different concentrations of ABA increased the contents of chlorophyll a, chlorophyll b, total chlorophyll and carotenoids in pea seedlings which were exposed to Cd^2+^ (Lu et al., 2018).

The retardation of photosynthesis in plants under Cd^2+^ stress has earlier been reported (Wang et al., 2013). Cd^2+^ stress decreases the rate of photosynthesis by interfering with the electron transport chain on the thylakoid membranes, the carbon cycle, and stomata conductance (Reddy, Chaitanya & Vivekanandan, 2004). In this study, Cd^2+^ treatment decreased the net photosynthesis (P_N_), stomata closure (Gs) and transpiration rate (Tr) but did not affect intercellular CO_2_ (Ci) of the leaves (Fig. 4). To a large extent, our results conform to an earlier report that indicated that higher Cd^2+^ concentrations decreased P_N_, Gs and Tr in lettuce plants (Dias et al., 2012). In comparison with the 100 µM CdCl_2_treatment, the application of ABA to the 100 µM CdCl_2_ treated plants also maintained higher P_N_, Gs and Tr. ABA has been referred to as ‘stress hormone’ and it protects plants against stress by inhibiting lipid peroxidation (Shen, Niu & Deng, 2017), regulating stomata movement (Pantin et al., 2012), enhancing the activities of antioxidant enzymes (Jiang & Zhang, 2001) and promoting photosynthesis (Gurmani et al., 2013). The results of our experiment is in conformity with a study which indicated that ABA at 10 µmol L^−1^ increased the P_N_, Gs, and Tr of lettuce plants under salt stress (Xie et al., 2018). Our results further showed that the application of ABA to the plants under CdCl_2_ stress protected the plants to some extent through the inhibition of lipid peroxidation (Fig. 1) and enhancement of the activities of antioxidant enzymes (Fig. 2). Moreover, ABA application maintained appreciable levels of photosynthetic pigments (Fig. 3) and promoted photosynthesis (Fig. 4) in the plants under CdCl_2_ treatment. Gurmani et al. (2013) also found that exogenous ABA promoted photosynthesis in *Oryza sativa* plants under salt stress. Previous study also indicated that different concentrations of Cd^2+^ did not affect the relative water contents (RWC) of the leaves in lettuce plants (Dias et al., 2012). However, our results showed that the exposure of the lettuce plants to 100 µM CdCl_2_greatly decreased the RWCs of the leaves. We observed that the transpiration rates of the plants under Cd^2+^ stress decreased (Fig. 4) and this probably reduced the root water uptake, leading to the low RWC of the leaves. The RWCs in the leaves of the plants under the Cd+ABA treatment was similar to that of the control plants, probably due to the role ABA plays in regulating stomata conductance.

The adverse effect of Cd^2+^ on the uptake and accumulation of nutrient elements in plants has been reported by several authors. For instance, in wheat seedlings, various concentrations of Cd^2+^ decreased the contents of essential trace elements especially Ca, Mg, K, Fe, Zn and Mn (Shukla et al., 2003). In the current experiment, we also found that with the exception of Cu, CdCl_2_ treatment decreased the contents of all the nutrient elements (Table 2). Cd^2+^ interferes with the uptake and transport of nutrient elements and decreases their contents in plants tissues (Hossain, Hasanuzzaman & Fujita, 2010). However, the contents of toxic Cd^2+^ in the leaves and roots of the Cd^2+^-treated plants were increased. Even though foliar application of ABA to the Cd^2+^-treated plants could not maintain the levels of the nutrient elements (except Fe) to levels as high as those in the control plants, it maintained the nutrient elements at levels greater than those in the sole CdCl_2_-treated plants (Table 2). In addition, the application of ABA to the CdCl_2_-treated plants resulted in Fe content similar to that in the control plants. The maintenance of higher levels of nutrient elements in the Cd+ABA treated plants could be attributed to the protection provided by ABA (decreased MDA and H_2_O_2_; increased chlorophyll and carotenoids) through the enhancement of the antioxidant enzymes defense system and promotion of photosynthesis. Han et al. (2016) reported that exogenous ABA application alleviated Cd^2+^ toxicity in *Populus euphratica* cells by restricting the uptake of Cd^2+^. In the present study, the contents of Cd^2+^ in the roots and leaves of the lettuce plants increased when the plants were grown in the nutrient solution which was supplemented with 100 µM CdCl_2_ (Fig. 6). A similar observation was made when lettuce plants were grown in nutrient solution supplemented with 10 mg L^−1^ Cd^2+^ (Tang et al., 2019). However, exogenous application of ABA to the 100 µM CdCl_2_ treated plants decreased the contents of Cd^2+^ in the leaves and roots of the plants compared with the sole 100 µM CdCl_2_ treated plants (Fig. 6). This observation could be attributed to the fact that exogenous ABA limited the uptake of Cd^2+^ by the roots and also restricted the root-to-leaf transfer of the absorbed Cd^2+^, maintaining the Cd^2+^ levels in the leaves within acceptable limits. Our results are similar to the findings of Tang and co-authors who reported that different concentrations of ABA improved the growth and decreased the content of Cd^2+^ in lettuce plants (Tang et al., 2019).

The biomass of several plant species and cultivars generally decrease under Cd^2+^ stress (Varalakshmi & Ganeshamurthy, 2009; Dong, Wu & Zheng, 2005). The results of our experiment also showed that the oxidative stress caused by Cd^2+^ culminated in decreased fresh and dry biomass of the roots and leaves of the lettuce plants (Table 2). We found that even though exogenous application of ABA to the plants under the Cd^2+^ stress could not maintain biomass at the same levels as in the control plants, the root and leaf biomass of the ABA treated plants were greater than sole CdCl_2_ treated plants and their biomass response to stress was also higher (Table 2). Thus, to a large extent, the exogenously applied ABA mitigated the Cd-induced stress and improved biomass accumulation in the lettuce plants. This observation could be attributed to the role ABA played in mitigating oxidative stress (inhibition of H_2_O_2_ accumulation) and promoting photosynthesis in the plants. Our result is in conformity with the findings of Wang and co-authors who indicated that the biomass of two ecotypes of *Solanum photeinocarpum* increased under Cd^2+^ treatment when exogenous ABA was applied (Wang et al., 2016).

In order to confirm the role of ABA in mitigating Cd^2+^ stress in the lettuce plants, we tested the effect of sole ABA and sole fluridone, which is an ABA-inhibitor, on the lettuce plants. Several reports have indicated that the application of high concentrations of ABA to unstressed plants causes oxidative stress and retards the growth and development of the plants (Hossain, Hasanuzzaman & Fujita, 2010; Li et al., 2010). However, in this experiment, the difference between sole ABA treated plants and the control plants was not significant for most of the indexes measured. This indicated that to a large extent, the exogenous ABA did not show any significant adverse effects on the growth of the lettuce plants in the absence of Cd^2+^ stress. This could be due to the low concentration (10 µg L^−1^) of the ABA applied in the current experiment. Our results collaborates the findings of Han et al. (2016) who reported that exogenous ABA reduced the effects of Cd^2+^ stress on the cells of *Populus euphratica* but had no obvious effects on the plants in the absence of Cd^2+^ stress. On the other hand, by interfering with ABA synthesis with the application of fluridone, the plants were obviously stressed. This was shown by the increased contents of H_2_O_2_ and MDA in the roots and leaves of the fluridone treated plants (Fig. 1). This observation was consistent with the findings of Hsu & Kao (2003) who reported that fluridone application increased the content of H_2_O_2_ in the leaves of a Cd-tolerant rice cultivar. Moreover, fluridone treatment also decreased the contents of chlorophyll and carotenoids (Fig. 3), inhibited photosynthesis by decreasing P_N_, Gs and Tr (Fig. 4) and also decreased the contents of nutrient elements in the leaves of the plants (Table 2). These results demonstrate that ABA actually play a crucial role in mitigating the Cd^2+^-induced stress and in improving the food safety of the Cd^2+^-treated lettuce plants by decreasing the content of Cd^2+^ in the leaves to levels within the acceptable.

## Conclusions

The results of our study have shown that exogenous ABA mitigated Cd^2+^-induced stress in Cd^2+^-sensitive lettuce genotype, *Lüsu*, by inhibiting lipid peroxidation (decreased H_2_O_2_ and MDA contents), increasing the activities of antioxidant enzymes (SOD, POD, CAT) and enhancing photosynthesis (increased P_N_, Gs and Tr). ABA application also increased the nutritional quality of the leaves by increasing the contents of essential nutrient elements (Mg, Ca, Fe, Mn, Zn). In addition, ABA application increased the safety of the leaves by decreasing the content of the toxic Cd^2+^ in the harvested leaves. This research has demonstrated that foliar application of appropriate concentrations of exogenous ABA can enhance Cd^2+^ tolerance and promote food safety of Cd^2+^-sensitive lettuce genotypes under conditions of Cd^2+^ pollution. It remains to be ascertained if similar results will be obtained under field conditions where soils or substrates are often used as growing medium for lettuce cultivation.

##  Supplemental Information

10.7717/peerj.9270/supp-1 Supplemental Information 1 Raw Data H2O2, MDA, SOD, POD, CAT, Chlorophyll content, Pn, Tr, Gs, Ci, RWC, Nutrient elements, Cadmium contents and plant biomass indexes, as influenced by the treatments.

## Additional Information and Declarations

### Data Availability

The following information was supplied regarding data availability: The raw data is available in a Supplementary File.
